# The roles of JAK2/STAT3 signaling in fusion of the secondary palate

**DOI:** 10.1242/dmm.050085

**Published:** 2023-10-17

**Authors:** Naoki Yoshida, Toshihiro Inubushi, Takumi Hirose, Gozo Aoyama, Hiroshi Kurosaka, Takashi Yamashiro

**Affiliations:** Department of Orthodontics and Dentofacial Orthopedics, Graduate School of Dentistry, Osaka University, Osaka 565-0871, Japan

**Keywords:** Cleft palate, Palatal fusion, JAK2/STAT3, p63, Folic acid

## Abstract

Cleft palate has a multifactorial etiology. In palatal fusion, the contacting medial edge epithelium (MEE) forms the epithelial seam, which is subsequently removed with the reduction of p63. Failure in this process results in a cleft palate. We herein report the involvement of janus kinase 2 (JAK2)/signal transducer and activator of transcription 3 (STAT3) signaling in palatal fusion and that folic acid rescues the fusing defect by reactivating JAK2/STAT3. In closure of bilateral palatal shelves, STAT3 phosphorylation was activated at the fusing MEE and mesenchyme underlying the MEE. JAK2 inhibition by AG490 inhibited STAT3 phosphorylation and resulted in palatal fusion failure without removal of the epithelial seam, in which p63 and keratin 17 (K17) periderm markers were retained. Folic acid application restored STAT3 phosphorylation in AG490-treated palatal explants and rescued the fusion defect, in which the p63- and K17-positive epithelial seam were removed. The AG490-induced palatal defect was also rescued in p63 haploinsufficient explants. These findings suggest that JAK2/STAT3 signaling is involved in palatal fusion by suppressing p63 expression in MEE and that folate restores the fusion defect by reactivating JAK2/STAT3.

## Teaser

**Summary:** Inhibition of JAK2/STAT3 results in failure to suppress p63, resulting in defects in palatal fusion. This defect is rescued by p63 haploinsufficiency and by folic acid treatment, reactivating JAK2/STAT3.

## INTRODUCTION

The Janus kinase (JAK)/Signal transducer and activator of transcription (STAT) signaling pathway is a central component that drives a variety of biological responses. More than 50 cytokines and growth factors have been identified in the JAK/STAT signaling pathway, which is involved in cellular proliferation, differentiation and organ development (Hu et al., 2021).

STAT3 is activated when the signal transduction protein JAK2 is stimulated by various cytokines and growth factors, such as epidermal growth factor (EGF), transforming growth factor (TGF)-α, platelet-derived growth factor (PDGF), hepatocyte growth factor (HGF), to phosphorylate STAT3. Evidence indicates that STAT3 is involved in cancer metastasis (Banerjee and Resat, 2016; Chang et al., 2013). For instance, cancer cell invasion is associated with increased STAT3 activation (Qin et al., 2019), and STAT3 activation decreases E-cadherin in colon cancers (Xiong et al., 2012).

The etiology of the nonsyndromic cleft palate is multifactorial, involving both genetic and environmental factors (Gritli-Linde, 2008), and recent genome-wide association studies and gene knockout animal experiments have identified many genetic variants (Alade et al., 2022). Genetic variants in *STAT3* have been found to be positively associated [nonspecific positive correlation (rs1905339, *P*=0.01; rs744166, *P*=0.02)] with nonsyndromic cleft lip and/or palate (Vieira et al., 2008). In addition, STAT3 heterozygote deficiency causes primary immunodeficiency hyper-IgE syndrome, and a cleft palate is observed in this syndrome (Grimbacher et al., 1999). However, little is known about how STAT3 affects the formation of the palate.

Our previous study demonstrated that STAT3 is involved in anterior palatal fusion in mice (Sarper et al., 2018). The palate consists of the primary and secondary palate, which make up the anterior and posterior portions, respectively (Levi et al., 2011). Molecular regulatory mechanisms in palatal fusion differ between the anterior and posterior parts of the palate (Bush and Jiang, 2012; Murray and Schutte, 2004). For example, loss of CBFB or RUNX1 results in cleft palate limited to the anterior region owing to defective palatal epithelial fusion. In these mutants, STAT3 phosphorylation is disturbed at the interface between the primary and secondary palates (Sarper et al., 2018). This anterior cleft is rescued by folic acid application in culture with the restoration of STAT3 phosphorylation (Sarper et al., 2019), indicating that STAT3 phosphorylation is involved in the palatal fusion process at the anterior palate. However, the role of JAK2/STAT3 in the secondary palate regions has not been elucidated.

Although the preventive effects of folic acid on facial clefts have been widely reported, the evidence is generally inconsistent (Wehby and Murray, 2010; Zhou et al., 2020), possibly owing to the diverse responses to folic acid supplementation in humans of different genetic backgrounds. Therefore, folate is not universally beneficial, and its effects may depend on genetic background. The molecular mechanisms involved in these effects remain poorly understood (Kappen, 2013).

In the development of the secondary palate, the fusion of medial edge epithelium (MEE) covering the bilateral palatal shelves generates an intervening epithelial seam, which is then removed so that the mesenchyme is continuous (Gaare and Langman, 1977; Trasler, 1968). Failure to remove this epithelial seam results in cleft palate or submucosal cleft palate.

p63 (*TP63*) is a homolog of the tumor suppressor p53 (*TP53*), and its mutation causes cleft palate in humans and mice, making it an important molecule for understanding the mechanism underlying the removal of the epithelial seam (Thomason et al., 2008; van Bokhoven and Brunner, 2002). p63 maintains the periderm on the oral tissues to prevent premature and pathological epithelial adhesion in early embryology (Richardson et al., 2014); however, overexpressed p63 disturbs the epithelial fusion in explanted palatal shelves in culture. The *Tgfb3* null mutation results in cleft palate owing to impaired removal of the MEE, and p63 immunoreactivity is retained in the unfused MEE. Interestingly, haploinsufficiency for p63 results rescues the cleft palate phenotypes in *Tgfb3* null mutants, indicating that the reduction in p63 expression is critical for the removal of the MEE during palatal shelf fusion (Richardson et al., 2017).

In the present study, we explored the roles of JAK2/STAT3 signaling in the development of the secondary palate using pharmaceutical application of AG490, a selective JAK2 inhibitor, and p63 haploinsufficient mice. We also showed that folic acid treatment restored STAT3 phosphorylation and had the potential to rescue the cleft phenotypes.

## RESULTS

### STAT3 phosphorylation in the developing secondary palatal shelves

**Fig. 1.**
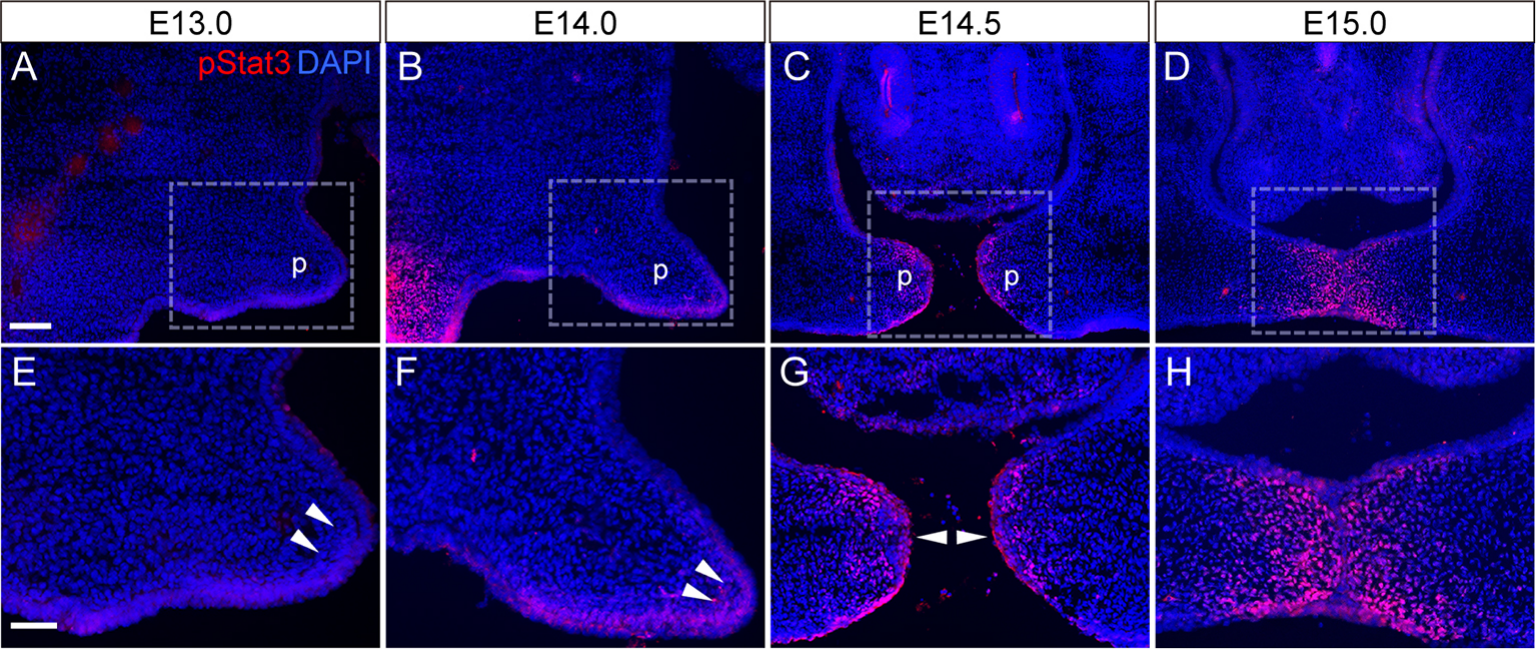
**Distribution of phosphorylated STAT3 (pSTAT3) in fusion of the secondary palatal shelves.** (A-D) Immunofluorescence analyses of pSTAT3 (red) in control mice. Nuclei were counterstained with DAPI (blue). (E,F,G,H) Higher-magnification views of the boxed areas in A, B, C and D, respectively. Arrowheads indicate pSTAT3 immunoreactivity at the tip of the secondary palatal process. The signals became more intense just before making contact with the bilateral palatal shelves (C,G). Scale bars: 100 μm (top), 40 μm (bottom). p, palatal shelves.

STAT3 activity was evaluated based on its phosphorylation state, and we performed immunohistochemical analyses of phosphorylated (p)STAT3. pSTAT3 immunoreactivity was detected at the tips of the palatal epithelium and underlying mesenchyme. Furthermore, the signals became more intense with the elongation of the secondary palatal shelves (Fig. 1A-H) and just before the contact of the bilateral palatal shelves (arrowheads in Fig. 1F,G). These findings suggested that STAT3 activity was activated by the epithelial fusion of the bilateral palatal shelves.

### Impairment of palatal fusion by the JAK2 inhibitor AG490

**Fig. 2.**
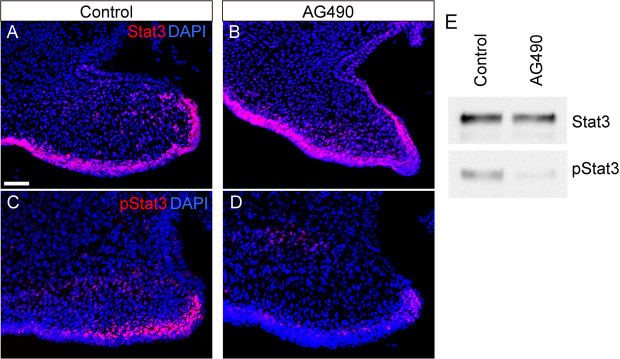
**Suppression of STAT3 phosphorylation by AG490.** (A-D) Immunofluorescence analyses of STAT3 (A,B, red) and pSTAT3 (C,D, red) in control (A,C) and AG490-treated (B,D) mice. AG490 downregulated pSTAT3 immunoreactivity (D). Nuclei were counterstained with DAPI (blue). (E) The downregulation of pSTAT3 immunoreactivity by AG490 treatment was confirmed by western blot analysis. Scale bar: 40 μm.

AG490 is a selective inhibitor of JAK2 that inhibits the phosphorylation of STAT3 in various cells including cancer cells (Yoshikawa et al., 2001). To evaluate the functions of STAT3 signaling in the development of the secondary palate, palatal explants were dissected at embryonic day (E)14.0 and treated with AG490 (Huang et al., 2016) in the suspension culture, as described previously (Takigawa and Shiota, 2004; Yamamoto et al., 2020). Immunohistochemical analyses (Fig. 2A-D) and western blotting (Fig. 2E) demonstrated that AG490 treatment attenuated pSTAT3 immunoreactivity, whereas STAT3 immunoreactivity was unaffected (Fig. 2A,B,E).

**Fig. 3.**
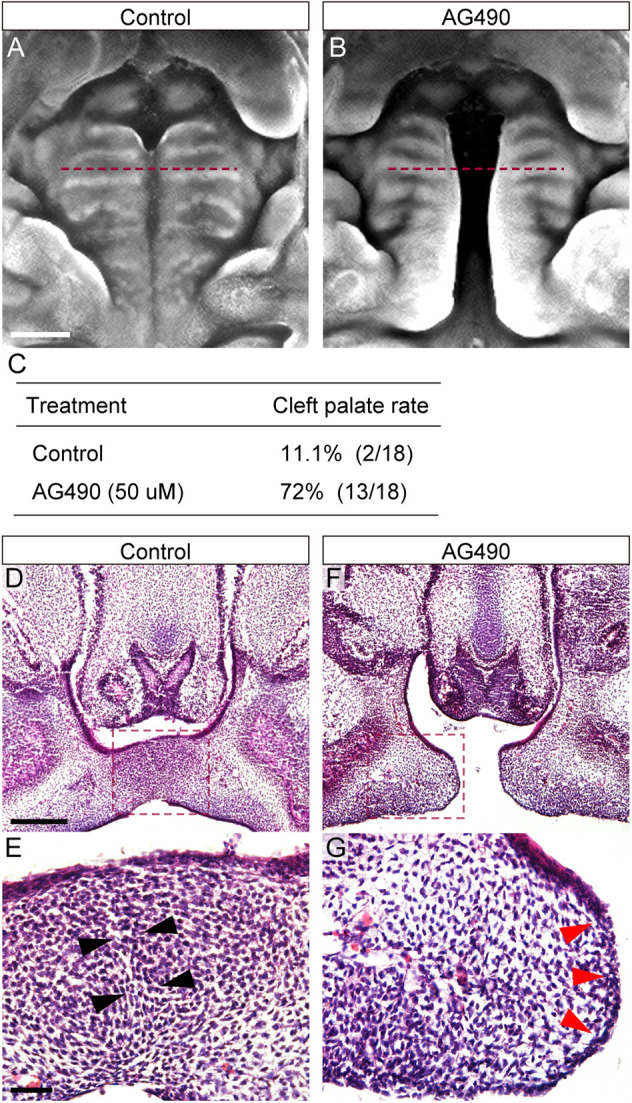
**Influence of JAK2 inhibition on palatal fusion in suspension culture.** (A,B) Occlusal views of explanted palates after 48 h of culture. The bilateral palatal shelves fused completely in palatal explants cultured in control conditions (A); AG490 treatment resulted in a cleft palate (B). Red dashed lines indicate the positions of histological sectioning. Scale bar: 1 mm. (C) Frequency of cleft palate in control and AG490-treated mice. AG490 treatment resulted in a cleft palate (13/18); cleft palate occurred less frequently in control animals (2/18) (C). (D-G) Histological sections of control (D,E) and AG490-treated (F,G) mice. (E,G) Higher-magnification views of the boxed areas in D and F, respectively. In control explants, the medial edge epithelium (MEE) was removed (black arrowheads in E). In AG490-treated explants, the palatal epithelium was retained at the tip of the contact regions (red arrowheads in G). Scale bars: 200 μm (top), 40 μm (bottom).

After 48 h of culture, AG490 treatment resulted in a cleft palate (13/18), whereas the palate fused in the controls (2/18) (Fig. 3A-C). Histological analysis confirmed that the MEE was removed in control mice, allowing for mesenchymal continuity (Fig. 3D,E, black arrowheads), whereas the epithelium was retained at the tip of the process in AG490-treated mice (Fig. 3F,G, red arrowheads).

### Characterization of the AG490-treated palatal epithelium in palatal fusion

To further investigate the cellular behavior of the medial edge of the secondary palate, the bilateral palatal shelves were dissected and placed on a modified Trowell system with a slight gap in the midline, which allowed the shelves to proliferate and fuse. In palatal fusion, the MEE terminates proliferation, thereby generating an intervening epithelial seam (Cuervo and Covarrubias, 2004; Cui et al., 2005). The periderms covering the fusing epithelium are sloughed off (Hu et al., 2015). The intervening epithelium then needs to be degraded in order to achieve mesenchymal confluence (Gritli-Linde, 2007).

**Fig. 4.**
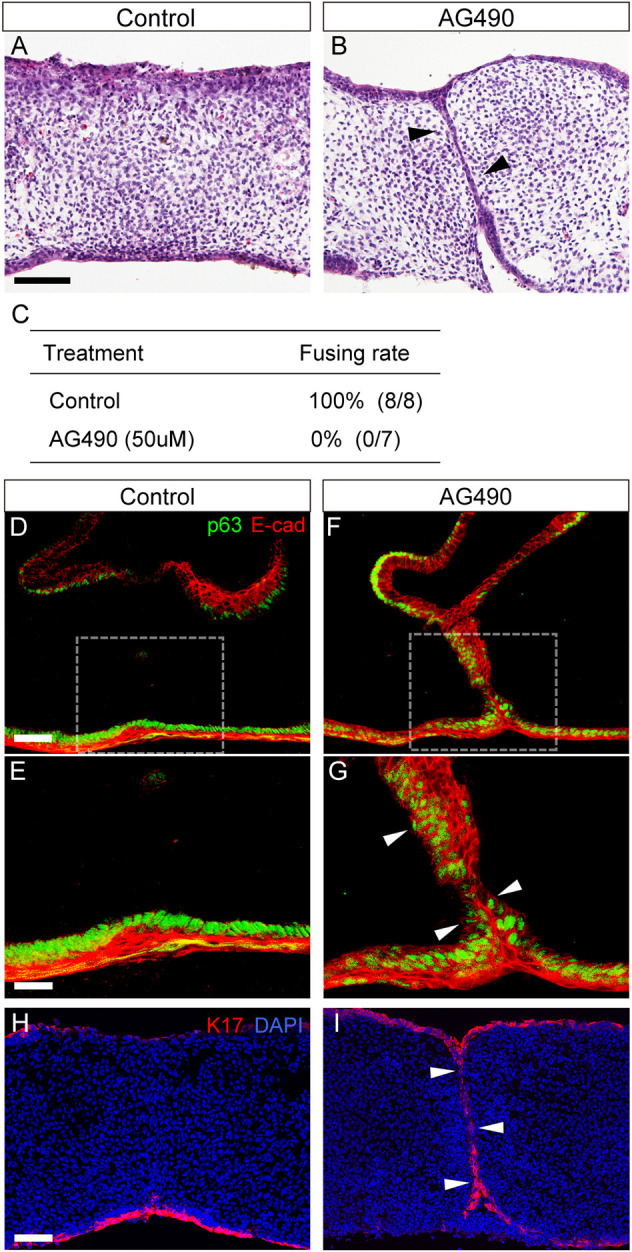
**Influence of JAK2 inhibition on removal of the epithelial seam in culture.** (A,B) Hematoxylin and Eosin staining confirmed the morphological differences between control and AG490-treated palates. (A) Control palates are completely fused. (B) In AG490-treated palates, the MEE (black arrowheads) persists in the midline of the secondary palate. (C) The fusing rate of secondary palatal shelves in control and AG490-treated mice. (D-G) p63 immunoreactivity in the MEE almost disappeared in the midline of the secondary control palate (D,E), whereas p63-immunoreactive MEE (white arrowheads in G) was retained in the AG490-treated palate (F,G). (E,G) Higher-magnification views of the boxed areas in D and F, respectively. (H) In controls, K17-immunoreactive periderm was sparsely observed in the epithelial remnants in the midline between the bilateral palatal shelves. (I) In AG490-treated palates, K17-immunoreactive periderms were retained at the epithelial seam (white arrowheads). Scale bars: 40 μm.

In our study, after 48 h of culture, our histological examination revealed that, in control mice, the MEE had disappeared throughout the entire palate in bilateral palatal shelves without any cleft (7/7; Fig. 4A,C). In AG490-treated mice, epithelial seams (black arrowheads in Fig. 4B) remained at the interface between the bilateral palatal shelves without any mesenchymal confluence (0/8; Fig. 4B,C).

In palatal fusion, downregulation of p63 in the MEE is essential for the removal of the fusing palatal epithelium (Iwata et al., 2013). Our immunohistochemical analysis revealed that p63-immunoreactive MEE disappeared at the midline between the bilateral palatal shelves in control mice (Fig. 4D,E), whereas p63 immunoreactivity was maintained in the unfused MEE in the AG490-treated mice (Fig. 4F,G). These findings suggested that JAK2 inhibition led to retained p63 expression, which was associated with failure in palatal fusion.

The periderm covers the fusing palatal process and is sloughed before palatal fusion (Hu et al., 2015). Keratin 17 (K17; KRT17) detects periderm (McGowan and Coulombe, 1998), and K17 immunoreactivity was sparsely observed in the epithelial remnants in the midline between the bilateral palatal shelves of E15.0 wild-type mice (Fig. 4H). However, in AG490-treated mice, K17-immunoreactive periderms were retained at the epithelial seam (white arrowheads in Fig. 4I).

**Fig. 5.**
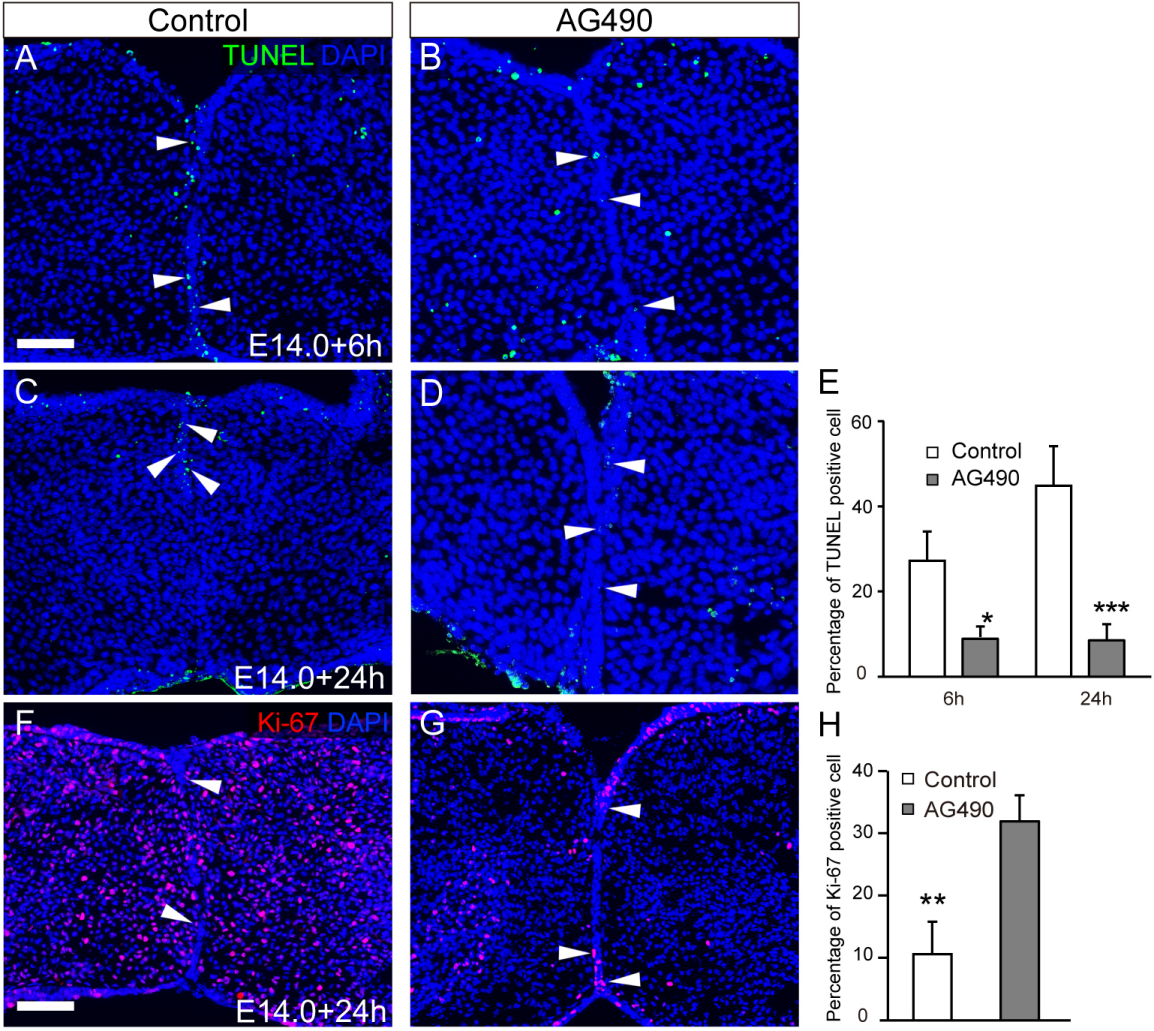
**Influence of JAK2 inhibition on apoptosis and proliferation in the epithelial seam.** (A-D) TUNEL-positive cells (green) were evident in the contacting and fusing MEE in E14 control palatal shelves at 6 h (A) and 24 h (C), respectively. In contrast, there were fewer TUNEL-positive cells in AG490-treated palatal shelves (B,D). (E) The percentage of TUNEL-positive MEE cells was significantly lower in the AG490-treated group than in the control group. (F,G) Ki67 signals (red) were sparse in the epithelial remnants in the control palates (F), whereas some Ki67-positive epithelium (arrowheads) was retained in the remnants of AG490-treated palates (G). (H) There were significantly more Ki67-positive epithelial cells in the AG490-treated palates than in the control palates. Nuclei were counterstained with DAPI (blue). Arrowheads indicate persistent periderm. *P<0.05, **P<0.01, ***P<0.01 (two-way ANOVA; data are shown as the mean±s.d.). Scale bars: 40 μm.

It has been established that, during epithelial fusion, the MEE ceases proliferation and undergoes apoptosis (Cuervo and Covarrubias, 2004; Cui et al., 2005). In the present study, terminal deoxynucleotidyl transferase dUTP nick-end labeling (TUNEL) staining revealed that, in control palate, TUNEL-positive cells were evident in the contacting and fusing MEE localized at the boundary between the bilateral secondary palate at 6 h (Fig. 5A) and 24 h (Fig. 5C), respectively, after the start of the culture. In contrast, there were fewer TUNEL-positive cells on the unfused MEE in the corresponding regions of the AG490-treated palate (Fig. 5B,D). The percentage of TUNEL-positive cells in the MEE cells was significantly lower in the AG490-treated group than in the control group (Fig. 5E).

The proliferative activity in the palatal epithelium was evaluated using Ki67 (MKI67) staining. Immunohistochemical analysis of Ki67 showed that Ki67-immunoreactive proliferating cells were retained in the epithelial remnants in AG490-treated mice, whereas Ki67-immunoreactive proliferating cells were sparsely present at the fused epithelium in control mice (Fig. 5F,G). There were significantly more Ki67-positive cells in the MEE in the AG490-treated group than in the control group (Fig. 5H).

Taken together, these findings show that AG490 treatment resulted in failed disintegration of the epithelial remnants with retention of proliferative activity, suppressed apoptosis and inadequate periderm removal, specifically at the junction between the secondary palatal shelves.

### Rescue of fusion defect by TGFβ3 treatment in AG490-treated mice

**Fig. 6.**
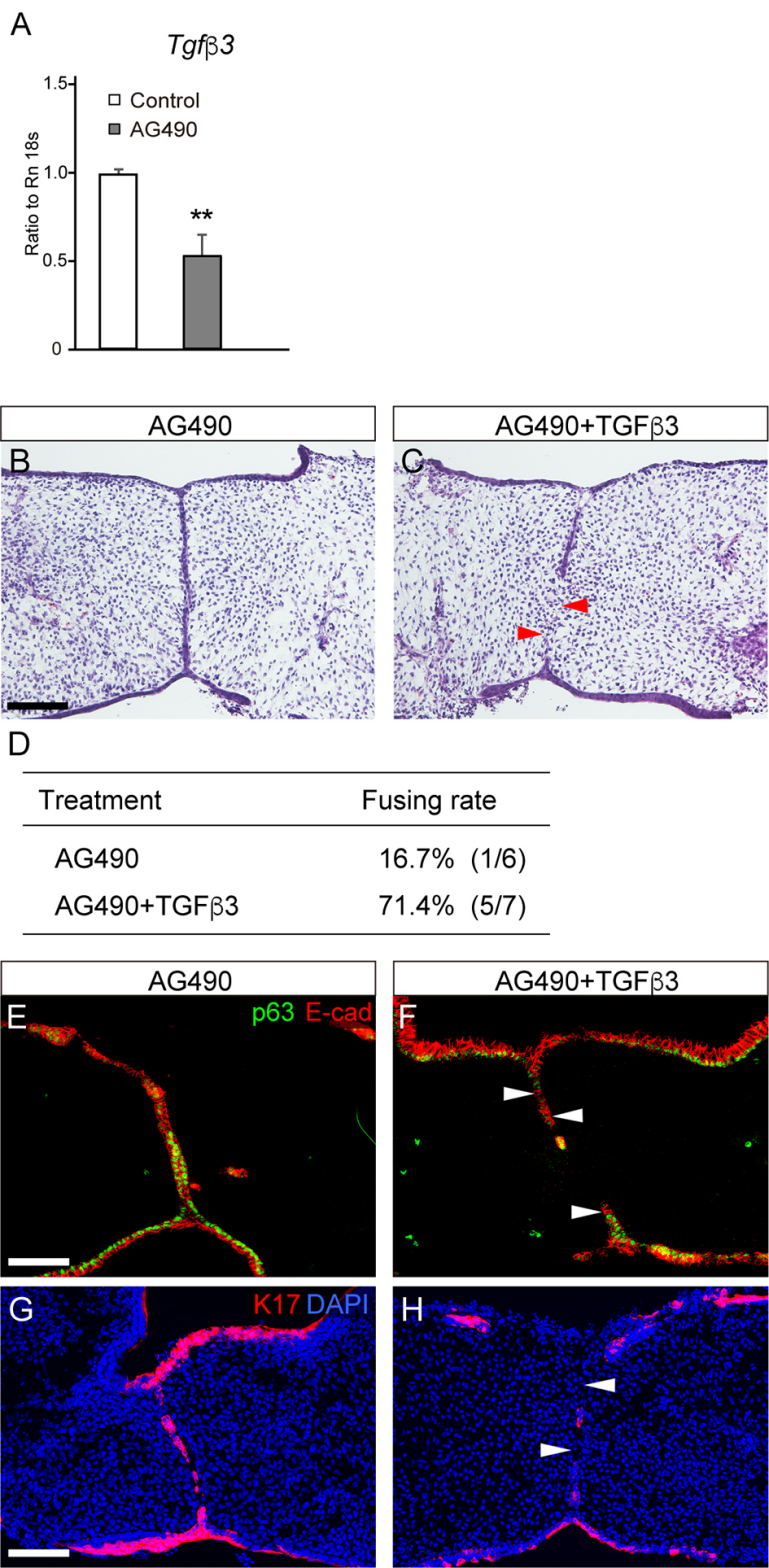
**Influence of JAK2 inhibition on *Tgfb3* expression in the microdissected epithelial seam and rescue of the JAK2 inhibition-induced palatal phenotypes by TGFβ3 treatment in culture.** (A) Quantitative PCR (qPCR) showed that *Tgfb3* expression was attenuated by approximately half in response to AG490 treatment. **P<0.01 (two-way ANOVA; data are shown as the mean±s.d.). (B,C) Histological sections demonstrating that failure in palatal fusion in AG490-treated palate was partially rescued by TGFβ3 application in culture. (D) The fusing rate of the palatal shelves in AG490-treated palates with and without TGFβ3 treatment. (E,F) p63 immunoreactivity in the MEE was also attenuated in the epithelial seam (white arrowheads) in response to TGFβ3 treatment in AG490-treated palatal explants. (G,H) Immunohistochemical analysis confirmed the removal of the retained K17-immunoreactive periderm in the epithelial seam (white arrowheads) in response to TGFβ3 treatment in AG490-treated palatal explants. Scale bars: 40 μm.

TGFβ signaling plays a crucial role in palatal fusion (Proetzel et al., 1995). Therefore, we first investigated the impact of AG490 treatment on *Tgfb3* mRNA expression and found that the expression of *Tgfb3* was reduced by approximately half (Fig. 6A). Then, we tested whether exogenous TGFβ3 protein could rescue AG490-treated palatal shelf fusion. The results revealed that exogenous TGFβ3 successfully rescued the fusion deficiency induced by AG490 administration (Fig. 6B-H). Considering the substantial downregulation of *Tgfb3* expression in response to AG490 treatment, this observation implies the presence of crosstalk between STAT3 signaling and TGFβ3 signaling, rather than the two signaling pathways functioning as independent pathways. It is important to note that reducing *Tgfb3* expression by approximately half cannot account for the cleft palate phenotypes observed in AG490-treated animals, as *Tgfb3* haploinsufficient mice do not develop cleft palate.

### Rescue of cleft palate by folic acid treatment in AG490-treated mice

Studies have shown that folic acid activates the STAT3 pathway (Wei et al., 2017; Hansen et al., 2015) and rescues the anterior cleft palate in *Cbfb* null mutant mice in culture (Sarper et al., 2019). Based on these findings, we investigated whether or not folic acid application could rescue the cleft palate caused by AG490 treatment with enhancement of STAT3 activity.

**Fig. 7.**
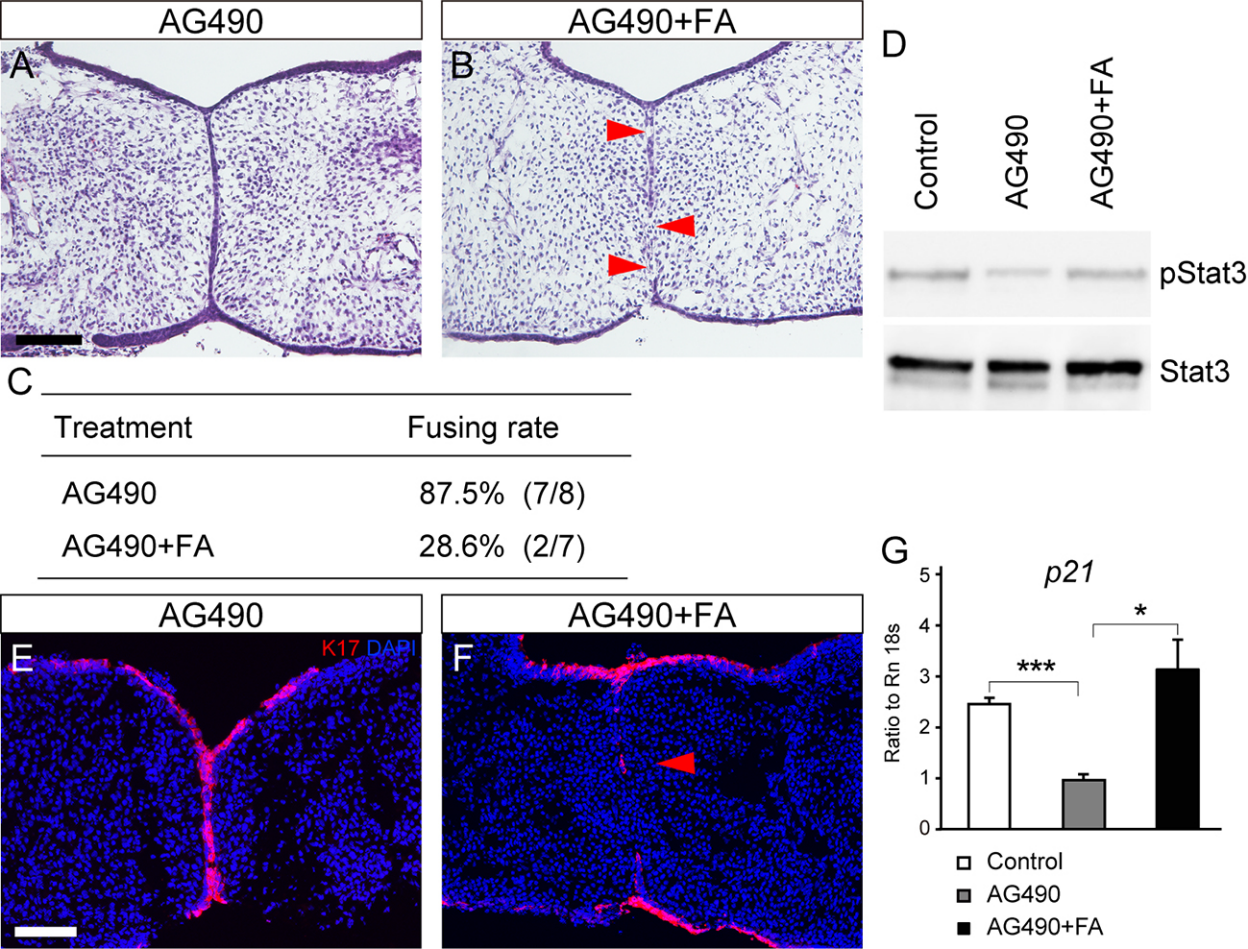
**Rescue of the JAK2 inhibition-induced palatal phenotypes by folic acid treatment in culture.** (A,B) Histological sections demonstrating that failure in palatal fusion in AG490-treated palate was partially rescued by folic acid (FA) application in culture (red arrowheads in B). (C) The fusing rate of the palatal shelves with folic acid treatment in AG490-treated explanted palates. (D) Western blot analysis confirmed that pSTAT3 immunoreactivity was upregulated by folic acid application. (E,F) Immunohistochemical analysis confirmed the removal of the retained K17-immunoreactive periderm in the epithelial seam (red arrowhead) by folic acid treatment in AG490-treated palatal explants. (G) qPCR showed that p21 expression was downregulated by AG490 treatment, whereas folic acid treatment restored p21 expression. *P<0.05, ***P<0.001 (two-way ANOVA; data are shown as the mean±s.d.). Scale bars: 40 μm.

After 48 h of treatment with folic acid in the Trowell culture, histological analysis demonstrated that folic acid application led to the removal of the epithelial seam and achievement of mesenchymal continuity in the AG490-treated palates in culture (Fig. 7A,B). The fusing rate in AG490-treated palates was 12.5% (7/8; Fig. 7C), whereas folic acid application rescued the failed palatal fusion to a fusing rate of 71.4% (5/7; Fig. 7C). Western blot analysis showed that folic acid application increased pSTAT3 immunoreactivity, as observed in cancer cells (Hansen et al., 2015). In contrast, STAT3 immunoreactivity was not altered (Fig. 7D). Immunohistochemical analysis also demonstrated that, in AG490-treated palatal explants, folic acid treatment successfully removed the K17-immunoreactive periderms in the epithelial seam (red arrowhead in Fig. 7F), whereas the epithelial seam was retained without folic acid treatment (Fig. 7E). These findings indicated that folic acid treatment restored the removal of the epithelial seam that was affected by JAK2 inhibition.

p21 (*Cdkn1a*) is a tumor suppressor gene and a cyclin-dependent kinase inhibitor that inhibits cell proliferation (Abbas and Dutta, 2009). In palatogenesis, p21 is essential for the removal of the epithelial seam of the secondary palate in the p63–p21 signaling axis, and p21 deficiency leads to the impairment of the fusion of the palatal shelves (Iwata et al., 2013). Quantitative polymerase chain reaction (qPCR) of the micro-dissected palatal epithelium showed that p21 expression was downregulated by AG490 treatment, whereas its expression was upregulated significantly by application of folic acid in AG490-treated palatal explants (Fig. 7G).

Taken together, these findings suggest that upregulated p21 might be associated with restored STAT3 activation in fusing palatal tissues.

### Rescue of AG490-induced cleft palate by reducing p63 expression in the MEE

In the present study, p63 expression was retained in the epithelial seam in AG490-treated explants, and previous studies have indicated that retained p63 is involved in cleft palate formation (Iwata et al., 2013; Richardson et al., 2017). Indeed, in a previous study, overexpression of p63 impaired removal of the epithelial seams in mouse palatal shelves in culture (Iwata et al., 2013).

**Fig. 8.**
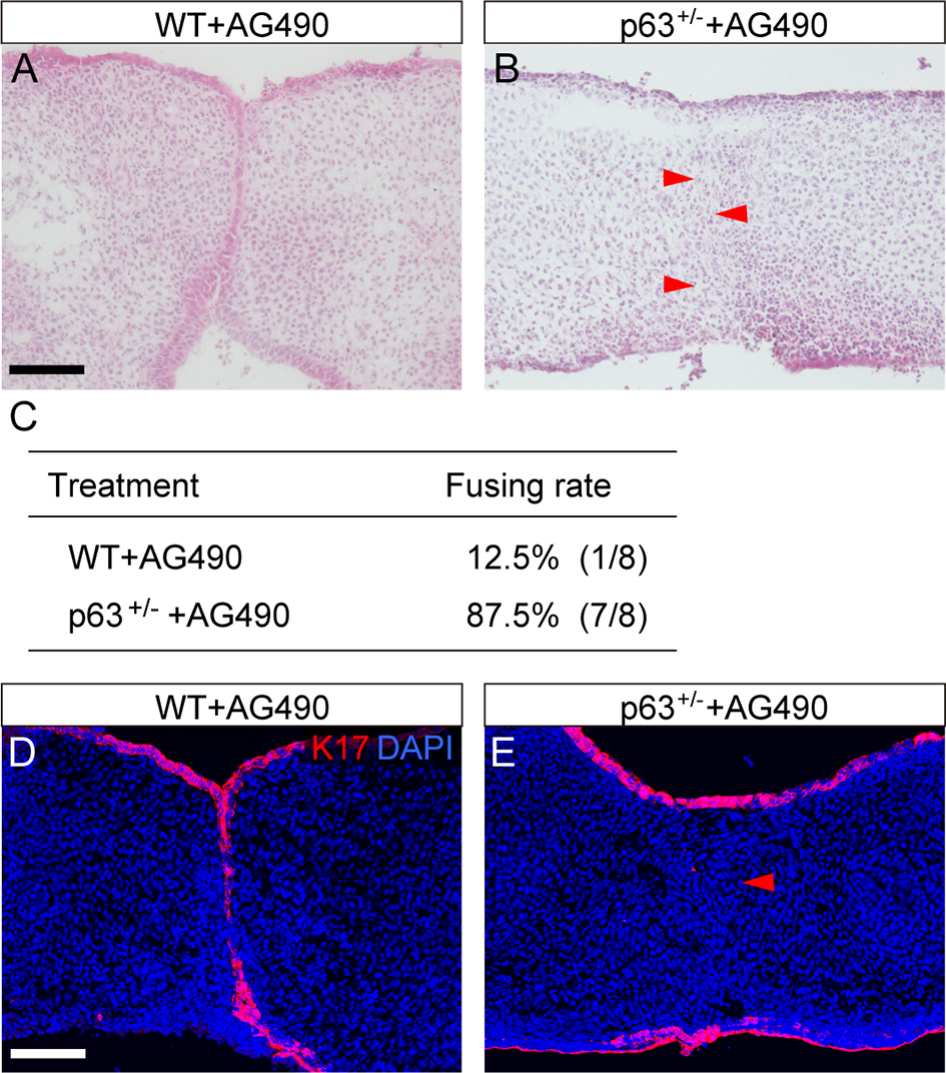
**Rescue of the JAK2 inhibition-induced palatal phenotypes by induction of p63 haploinsufficiency.** (A,B) Histological sections demonstrating that failure in palatal fusion in AG490-treated mice was rescued by reducing p63 expression in the MEE. The epithelial seam was removed in p63^+/−^ palate (red arrowheads in B). (C) The fusing rate of the palatal shelves in AG490-treated explanted palates with p63 haploinsufficient mutation. (D,E) Immunohistochemical analysis confirmed the removal of the retained K17-immunoreactive periderm in the epithelial seam (red arrowhead) by induction of p63 haploinsufficiency in AG490-treated palatal explants. Scale bars: 40 μm.

We therefore evaluated the influence of reduction in p63 expression on the removal of the epithelial in the AG490-treated palate. Although AG490 treatment led to the retention of the epithelial seam at the contact regions of the bilateral palatal shelves (Fig. 8A), the epithelial seam was removed in p63 haploinsufficient mice (red arrowheads in Fig. 8B). The fusing rate with AG490-treated palates was 12.5%, and p63 haploinsufficiency rescued the failed palatal fusion, with a fusing rate of 87.5% (Fig. 8C). Immunohistochemical analysis confirmed that K17-positive periderm was removed in p63 haploinsufficient mice with AG490 treatment (Fig. 8D,E).

## DISCUSSION

This is the first time that the role of the JAK2–STAT3–p63 axis in development of the secondary palate has been explored. Dysfunction of this signaling axis may play some role in cleft palate, specifically in the fusion of the bilateral palatal shelves. Cleft palate was characterized by impaired removal of the epithelial seam, and the fusion defect was rescued by restoration of STAT3 phosphorylation using folic acid application and the introduction of p63 genetic haploinsufficiency. Thus, our findings suggested the function of JAK2/STAT3 in palatal fusion and also provided experimental basis for the beneficial use of folic acid for cleft palate.

STAT3 mutations underlie nonspecific cleft palate (Grimbacher et al., 1999). The JAK2/STAT3 pathway is involved in a wide range of cellular functions, and aberrations in various upstream activator genes of JAK2/STAT3 signaling may lead to inhibition of STAT3 activation in cleft palate (Miettinen et al., 1999; Sarper et al., 2018). Thus, in addition to STAT3 mutations, various genetic mutations upstream of JAK2/STAT3 signaling may cause dysfunction of JAK2/STAT3 signaling, resulting in cleft palate. Such potential upstream genes include platelet-derived growth factor receptor alpha/beta (*Pdgfra/b*); epidermal growth factor receptor (*Egfr*); transforming growth factor alpha (*Tgf*); connective tissue growth factor (*Ctgf*; *Ccn2*); fibroblast growth factor receptor 1 and 2 (*Fgfr1/2*); fibroblast growth factor 9, 10 and 18 (*Fgf9/10/18*); *Wnt5a/7a*; and *Tgfb1/2/3* (Ozturk et al., 2013). It is expected that future studies will clarify the relationship between STAT3 signaling and the cleft palate-causing genes mentioned above.

Our study findings provide a new experimental model to explore the mechanisms underlying fusion and the pathogenesis of fusion disorders in cleft palate. We found that AG490 inhibits STAT3 phosphorylation, suppresses epithelial seam removal at the contact surface of the palatine process and inhibits fusion of the palatine process. Furthermore, our experimental model reveals at least some of the molecular mechanisms by which folic acid prevents the development of cleft palate. A similar approach could be used to explore molecules that prevent cleft palate in the future.

The involvement of p63 in MEE fusion provides clues on how the JAK2/STAT3 pathway regulates the integration of MEE. Previous studies have shown that the downregulation of p63 expression, which promotes periderm migration and suppresses MEE proliferation, is essential for palatal fusion (Richardson et al., 2017). In our study, the failure of palatal fusion due to JAK2 inhibition was also characterized by retention of p63 immunoreactivity in the persisting MEE. This palatal phenotype was rescued by the induction of p63 haploinsufficiency in AG490-treated mice. Thus, our findings indicate that the JAK2/STAT3 pathway regulates epithelial seam removal by regulating p63.

TGFβ signaling plays a crucial role in palatal fusion (Proetzel et al., 1995). In our study, *Tgfb3* expression was reduced by approximately half in AG490-treated palates (Fig. 6A). Furthermore, exogenous TGFβ3 successfully rescued the fusion deficiency induced by AG490 administration (Fig. 6B-H). Considering the substantial downregulation of *Tgfb3* expression in response to AG490 treatment, this observation implies the presence of crosstalk between STAT3 and TGFβ3 signaling in palatal fusion, rather than the two signaling pathways functioning as independent pathways.

**Fig. 9.**
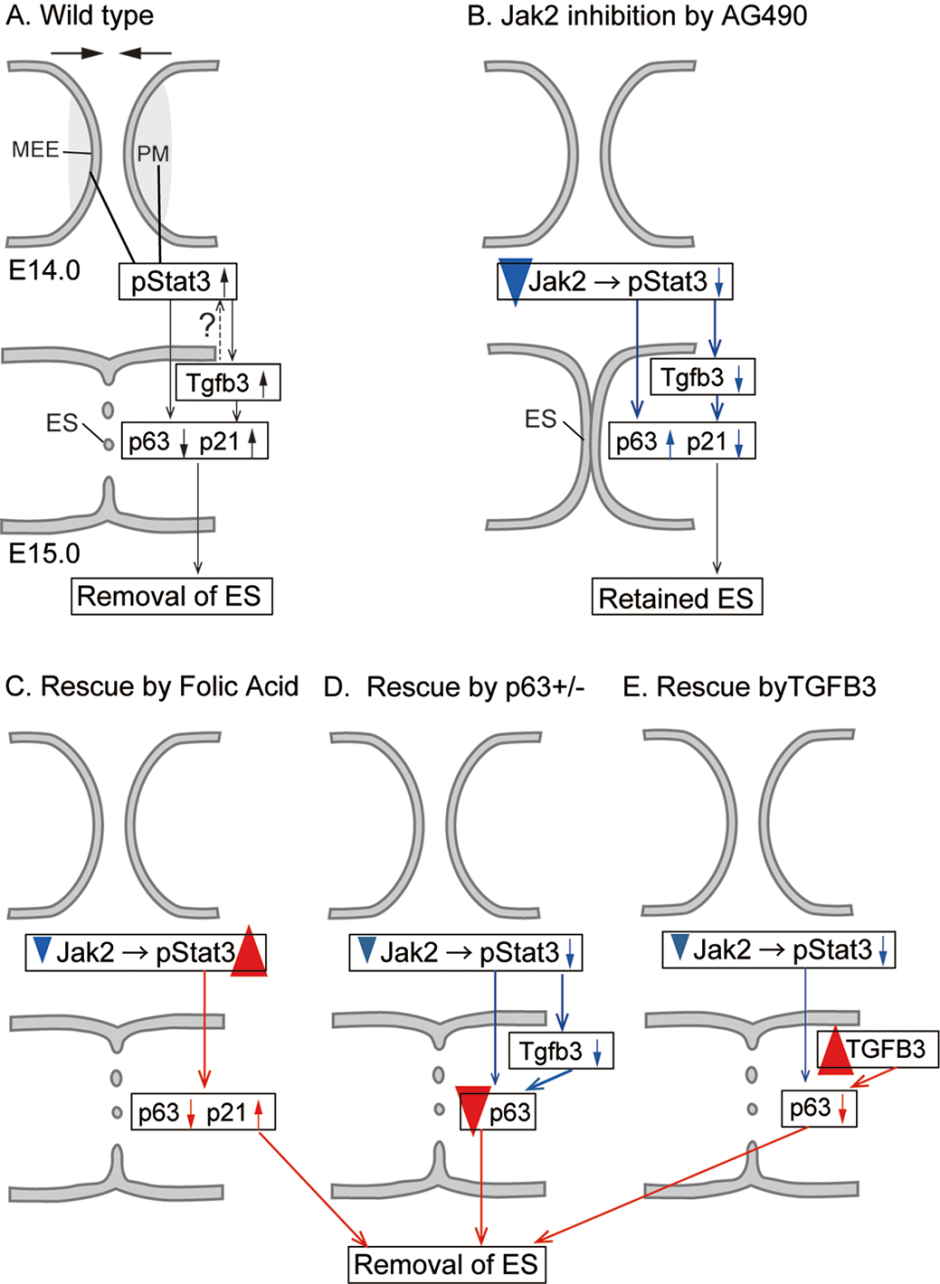
**Schematic of the key findings of this study.** (A) At the tip of the fusing palatal process, STAT3 phosphorylation is activated during palatal shelf elongation. After the palatal processes come into contact, the MEE forms the epithelial seam, which is subsequently removed, with reduction of p63 and upregulation of p21. (B) STAT3 phosphorylation is suppressed by JAK2 inhibition, which inhibits the removal of the epithelium seam, with retention of p63 and downregulation of p21. (C) Folic acid treatment activates phosphorylation of STAT3 in JAK2 inhibitor-treated explants, and the epithelial seam is removed, with upregulation of p21. (D) In p63 haploinsufficient explants, treatment with JAK2 inhibitors eliminates epithelial seams. (E) In TGFβ3-treated explants, the epithelial seam is removed, with reduction of p63. ES, epithelial seam; MEE, medial edge epithelium; PM, palatal mesenchyme underlying the MEE.

However, crosstalk between TGFβ and STAT3 signaling should be considered for understanding AG490-induced palatal fusion failure and its rescue by the introduction of p63 haploinsufficiency. Retained expression of p63 in the fusing epithelium could be causative of fusion failure in AG490-treated mice, as well as in *Tgfb3* null mutant mice. Although *Tgfb3* expression was reduced by ∼50% in AG490-treated mice, cleft palate does not occur in *Tgfb3* heterozygous mice. Therefore, the reduction in *Tgfb3* does not solely cause palatal fusion defects. Considering that AG490-induced fusion defect was rescued by TGFβ3 protein in this study, it is likely that TGFβ3 signaling functions to downregulate p63 under AG490 treatment. Furthermore, a possible molecular mechanism by which p63 heterozygosity rescued palatal fusion defects caused by AG490 treatment could be a dose-dependent interaction between this AG490-decreased *Tgfb3* and reduced p63 (Fig. 9E).

p21 is also involved in the removal of the epithelial seam. p21 is specifically expressed in palatal ectoderm, and its overexpression restores the degeneration of the MEE due to *Tgfbr2* deficiency in culture, indicating its essential role in palatal fusion (Iwata et al., 2013). In the present study, JAK2 inhibition led to the downregulation of p21 expression, and folic acid upregulated STAT3 phosphorylation and p21 mRNA expression. JAK2 inhibition-induced downregulation of p21 was restored in p63^+/−^ explants. Thus, this and previous studies (Iwata et al., 2013) suggest that the JAK2/STAT3 pathway regulates the removal of the epithelial seam by regulating p63 and p21 expression.

The role of JAK2/STAT3 signaling in palatogenesis was further supported by our rescue experiment using folic acid, in which folic acid upregulated STAT3 phosphorylation and rescued the impaired palatal fusion caused by AG490 treatment. Neural tube defects are also caused by failure in fusion of the neural folds and neural plates (Greene and Copp, 2014). In neural tube development, STAT3 is phosphorylated in the ectoderm at the edge of the neural plates (Shafique and Winn, 2020), and decreased STAT3 activity is associated with impaired plate fusion. Folic acid intake has been shown to prevent the occurrence of neural tube defects (De-Regil et al., 2015; Obican et al., 2010). Interestingly, folic acid binds to folate receptor α (FRα; FOLR1) and mediates STAT3 activation in a JAK-dependent manner, which explains the involvement of FRα–STAT3 signal transduction in the role of folic acid in preventing neural tube defects (Hansen et al., 2015). These findings suggest a promising molecular mechanism underlying folic acid application and provide insights into the prevention of cleft palate.

Although preventive effects of folic acid on orofacial clefts are commonly reported, the evidence is generally inconsistent (Wehby and Murray, 2010; Zhou et al., 2020), and the molecular mechanisms involved in these effects remain poorly understood. Our study results suggest that folic acid is beneficial only if the impaired palatal fusion is due to dysfunction of the JAK2/STAT3 axis and that such an effect depends on genetic configuration. This could be why folic acid is not universally beneficial and shows diverse effects. It is also likely that efforts to prevent cleft palate via folic acid application may need to be more specifically targeted. Cleft palate is formed during the embryonic stage of pregnancy. Further investigation to identify individuals with an increased risk of preventable cleft palate is warranted, and mouse models are indispensable for improving our understanding of the JAK2/STAT3 axis.

One limitation of this study is that AG490 was used as an inhibitor of the JAK2/STAT3 pathway; many kinase inhibitors often have off-target effects on the enzymes they target. AG490 is relatively specific to JAK2; however, there might be potential for compensation or redundancy among JAK/STAT pathways. Our study results confirmed that AG490 was able to markedly downregulate STAT3 activity, specifically in the fusing palatal epithelium, as indicated by decreased STAT3 phosphorylation levels at concentrations of 50 μM. We also confirmed that pSTAT1 and pSTAT5 were scarcely detected.

Another limitation concerns the tissue-specific role of pSTAT3 activation. In the present study, pSTAT3 activation was found not only in epithelium but also in underlying mesenchyme. However, in the palatal process, expression of p21 and p63 was restricted to the epithelium, in contrast to pSTAT3. The expression of p21 and p63 has been reported to be regulated downstream of STAT3 using cancer cells and cell lines (Ma et al., 2010; Sinibaldi et al., 2000). Thus, it is possible that p21 and p63 changes associated with STAT3 phosphorylation are mediated by epithelial STAT3 signaling. However, STAT3 has multiple roles and STAT3 actions are context dependent, as shown in a study using cancer cells (D'Amico et al., 2018). In the present study, pSTAT3 was found to be increased in the mesenchyme underlying the MEE. Therefore, we cannot exclude the possibility that the expression of some mesenchymal signaling molecule is correspondingly enhanced and indirectly regulates the expression of p63 and p21 in the epithelium by signals from the mesenchyme. Future analyses using animals with tissue-specific genetic modification of the palatal epithelium and mesenchyme are needed to clarify this point.

In conclusion, our findings provide new evidence of JAK2/STAT signaling involvement in the fusion of the secondary palate and the genetic etiology of the cleft palate. In secondary palatal development, STAT3 was specifically activated at the fusing epithelium of the bilateral palatal shelves (Fig. 9A). JAK2 inhibition by AG490 downregulated STAT3 phosphorylation and impaired epithelial fusion, with retained p63-immunoreactive epithelial seam and downregulated p21 expression, indicating that JAK2/STAT3 signaling is involved in the removal of the epithelial seam (Fig. 9B). This finding was supported by rescue of the impairment of palatal fusion using folic acid, which restored STAT3 phosphorylation, and the residual epithelial seam being removed (Fig. 9C). JAK2 inhibitor-induced failure in palatal fusion was rescued in p63^+/−^ mice, supporting the notion that the JAK2/STAT3 pathway is involved in the downregulation of p63 in palatal fusion (Fig. 9D). Furthermore, TGFβ3 protein rescued the JAK2 inhibitor-induced failure in palatal fusion, suggesting that there is a crosstalk between TGFβ3 and STAT3 signaling in the regulation of p63 in palatal fusion (Fig. 9E).

## MATERIALS AND METHODS

### Study approval

All animal experiments were performed in strict accordance with the guidelines of the Animal Care and Use Committee of the Osaka University Graduate School of Dentistry, Osaka, Japan. The protocol was approved by the Committee on the Ethics of Animal Experiments of Osaka University Graduate School of Dentistry. Mice were housed in the animal facility at the Department of Dentistry, Osaka University. Procedures were performed with the approval of the Osaka University Graduate School of Dentistry Animal Committee and adhered to welfare guidelines.

### Animals

Experiments were performed on mature female ICR (Japan SLC) and C57BL/6J (CLEA Japan) mice. Genotyping of the BALB/c p63 mouse line has been described previously (Thomason et al., 2008). Embryos were dissected from time-mated pregnant female mice, and the day on which a vaginal plug was found was designated as day 0 of pregnancy. Time-course observation of palatal shelf development was performed by dissecting the mouse maxilla from E13.0 to E14.5.

### Suspension culture

Embryo heads from E13.5 ICR mouse embryos were collected in BGJb medium (Gibco), and the mandibles, tongues and brains were removed. The remaining palatal tissue, including the primary and secondary palate and the nasal septum, was cultured for 48 h in a whole-embryo culture incubator (RKI Ikemoto) at 37°C. Palatal tissues were incubated in BGJb medium with or without AG490. Tissues were harvested after 48 h of culture.

### Dissection and organ culture

On E13.5, wild-type ICR mouse embryos were quickly immersed in BGJb medium. As previously reported (Inubushi et al., 2022), the palatal shelves were removed using forceps under a dissecting microscope. The modified Trowell system was used for culture. Isolated palatal shelves were placed in pairs on 0.4-μm porosity filters (MilliporeSigma), nasal epithelium down, media edges in contact, on 35-mm tissue culture dishes (Corning). The culture medium was composed of BGJb medium with or without AG490 (658411; Sigma-Aldrich), or with AG490 and folic acid (Nacalai Tesque). Samples were pretreated for 6 h before organ culture. Palatal shelves were cultured at 37°C with 5% CO_2_.

### Application of TGFβ3 protein in palatal explants in culture

Affi-Gel beads (Bio-Rad) were incubated in TGFβ3 (100 ng/μl, R&D Systems), or bovine serum album for controls, and placed between the edges of dissected palatal shelves (Sarper et al., 2018).

### Assessment of palatal fusion and histological analysis

The palatal phenotypes were first evaluated under a dissecting microscope. For histology, dissected samples were fixed in 4% paraformaldehyde, equilibrated in graded sucrose and embedded in Tissue-Tek (OCT compound; Sakura, Japan). The tissue samples were sectioned into 10-μm slices.

### Immunohistochemistry and TUNEL staining

Immunofluorescence staining was performed on 15-μm sections using monoclonal rabbit anti-STAT3 (1:200; 9139, Cell Signaling Technology), monoclonal rabbit anti-pSTAT3 (1:200; 9145, Cell Signaling Technology), monoclonal mouse anti-p63 (1:200; sc-25268, Santa Cruz Biotechnology), polyclonal rabbit E-cadherin (1:200; ab15148, Abcam) or polyclonal rabbit anti-Ki67 (1:400; ab15580, Abcam) overnight at 4°C. Alexa Fluor 546-conjugated donkey anti-mouse IgG (1:400; A10036, Thermo Fisher Scientific), Alexa Fluor 488-conjugated donkey anti-mouse IgG (1:400; A21202, Thermo Fisher Scientific) or Alexa Fluor 555-conjugated goat anti-mouse IgG (1:400; A21428, Thermo Fisher Scientific) was then used as a secondary antibody. The sections were counterstained with 4′,6-diamidino-2-phenylindole (DAPI; 1:500; Dojindo) and mounted in fluorescent mounting medium (Dako). At least three embryos of each genotype were used for each analysis. Apoptotic cells were identified using an *in situ* cell death detection kit (11684795910; Roche), according to the manufacturer's instructions. In the TUNEL assay, cells that exhibited distinct nuclear fluorescence were identified as apoptotic and were counted.

### Immunoblotting

Protocols for immunoblotting were as described previously (Inubushi et al., 2022). In brief, cells were lysed in ice-cold RIPA buffer containing 50 mmol/l Tris-HCl (pH 7.6), 150 mmol/l NaCl, 1% Nonidet P40 Substitute, 0.5% sodium deoxycholate and 0.1% sodium dodecyl sulfate (SDS). A protease inhibitor cocktail was purchased from Promega. Following a 30-min lysis period on ice, lysis samples were centrifuged at ∼20,000 ***g*** for 20 min at 4°C to prepare cell lysates. A total of 10 μg lysate was then subjected to SDS-polyacrylamide gel electrophoresis (PAGE) on an 8-16% Tris-glycine gel (Invitrogen), followed by electroblotting onto an Immobilon PVDF membrane (EMD Millipore). ECL Western Blotting Substrate (07880; Nacalai Tesque) was used to detect signals. The following antibodies were used: anti-STAT3 (1:1000; 9139, Cell Signaling Technology), anti-pSTAT3 (1:1000; 9145, Cell Signaling Technology), anti-α-tubulin (1:1000; T6074, Sigma-Aldrich), horseradish peroxidase-conjugated goat anti-rabbit IgG (1:500; 1706565, Bio-Rad) and horseradish peroxidase-conjugated goat anti-mouse IgG (1:500; 1706515, Bio-Rad).

### Laser microdissection

Mouse embryonic maxilla after organ culture was freshly embedded in OCT compound and frozen immediately. Tissues were serially sectioned at −20°C on a cryostat (CM 1950, Leica) at a thickness of 25 μm. The maxilla was sectioned from the anterior to the posterior direction throughout the palate until reaching the secondary palate. The tissue sections were mounted and thawed on a film-coated slide. In total, there were 24-26 serial sections obtained from the secondary palate at E14.5 (section numbers varied owing to the orientation of the frozen block). The MEE of the palatal explants was dissected from the sections using a Leica Micro Laser System (LMD6500) and collected by tube.

### RNA extraction and qPCR analyses

The E13.5 palatal shelves were incubated with or without AG490 or with AG490 and folic acid for 48 h. After culture, we dissected only the MEE region using laser microdissection. Protocols for RNA extraction and qPCR were as described previously (Inubushi et al., 2022). First, total RNA was extracted from the dissected tissues using IsogenII (Nippon Gene) according to the manufacturer's protocol. The RNA was then reverse transcribed to cDNA using oligo (dT) with reverse transcriptase (Takara). For real-time PCR, aliquots of total cDNA were amplified with TaqMan Fast Universal PCR Master Mix (Applied Biosystems). Data acquisition and analyses were performed with a Step One Real-Time PCR System using the Step One software program, Version 2.1 (Applied Biosystems). The PCR products were quantified using *Gapdh* as the reference gene. The primers and TaqMan probes were purchased from Applied Biosystems.

### Statistical analyses

Statistical methods were not used to predetermine the sample size. Statistical analyses were performed with the GraphPad Prism 8 software program. Unpaired two-tailed Student's *t*-test and two-way ANOVA were used under the assumption of a normal distribution and observance of similar variance. *P*<0.05 was considered significant. A Bonferroni post hoc analysis was performed where applicable. Values are expressed as the mean±s.d. For all experiments, variances between groups were similar, and data were symmetrically distributed. The data shown are representative images; each analysis was performed on at least three mice per genotype. Immunostaining was performed at least in triplicate.
